# Standardised Transplant-Orientated Kasai Portoenterostomy in a Combined Kasai and Transplant Programme: An 11-Year Single-Surgeon Series of 74 Consecutive Cases

**DOI:** 10.3390/jcm15166275

**Published:** 2026-08-13

**Authors:** Fahim Kanani, Abed Elrahman Dahly, Raymond Reding, Aviad Gravetz, Orith Waisbourd-Zinman, Yael Mozer-Glassberg, Chaya Shwaartz, Eviatar Nesher, Michael Gurevich

**Affiliations:** 1 Department of Abdominal Organs Transplantation, Rabin Medical Center—Beilinson Campus, Sackler Faculty of Medicine & Health Sciences, Tel Aviv University, Tel Aviv 6997801, Israel; 2 Pediatric Liver Transplant Program, Cliniques Universitaires Saint-Luc, Université Catholique de Louvain, 1200 Brussels, Belgium; 3 Department of Pediatric Gastroenterology, Nutrition and Liver Diseases, Schneider Children’s Medical Center of Israel, Petah Tikva 4920235, Israel; 4 Gray Faculty of Medical and Health Sciences, Tel Aviv University, Tel Aviv 6997801, Israel; 5 Department of Transplantation and HPB Surgery, Toronto General Hospital, Toronto, ON M5G 2C4, Canada; 6 Department of Pediatric Surgery, Schneider Children’s Medical Center of Israel, Petah Tikva 4920235, Israel

**Keywords:** biliary atresia, portoenterostomy, hepatic, liver transplantation, treatment outcome, surgical procedures, operative

## Abstract

**Background**: Kasai hepatoportoenterostomy (KPE) remains first-line therapy for biliary atresia(BA), yet the majority of patients will ultimately require liver transplantation. In centres where both KPE and paediatric liver transplantation are performed, KPE has been understood since the early 2000s as the first stage of a two-stage strategy, and the individual technical elements described here are established practice. What is reported is their uniform application as a single written protocol from the first case of the series, together with native liver and transplant outcomes in the resulting cohort. The technical conduct of KPE directly influences the safety and complexity of subsequent transplantation, yet operative decisions at the time of KPE have rarely been evaluated from a transplant-optimisation perspective. We describe a standardised KPE approach incorporating three technical modifications intended to preserve favourable conditions for eventual hepatic replacement and report native liver and transplant outcomes in the resulting cohort. **Methods**: A retrospective analysis was conducted of 74 consecutive KPE procedures performed by a single surgeon between 2014 and 2025 at Schneider Children’s Medical Center, Israel. The operative approach incorporated three deliberate modifications applied uniformly from the first case: a transverse subcostal incision aligned with future transplant access, avoidance of liver mobilisation and exteriorisation, and standardisation of the Roux limb at 50 cm. Primary outcomes were native liver survival and transplant operative parameters. **Results**: Of the 74 patients, 39 (52.7%) maintained their native liver throughout follow-up, while 35 (47.3%) required liver transplantation. No peri-operative mortality occurred. Median age at KPE was 53 days. In the Cox model, post-KPE portal hypertension (adjusted hazard ratio (aHR) 3.63, 95% confidence interval (CI) 1.40–9.42, *p* = 0.008) and hepatopulmonary syndrome (adjusted HR 10.23, 95% CI 2.19–47.85, *p* = 0.003) were associated with eventual transplantation; complications were modelled as fixed (ever/never) covariates because onset dates were not consistently retrievable, and these estimates may be subject to immortal-time bias. Among transplanted patients, the median operative time was 8.0 h (interquartile range (IQR) 6.0–10.2) (*n* = 34); intraoperative blood loss was documented in 15 of 35 patients (median 300 mL (IQR 205–450)). **Conclusions**: A standardised, transplant-orientated KPE approach was applied uniformly across 74 consecutive cases; 52.7% maintained their native liver, and transplantation in the remainder proceeded without peri-operative mortality. This series was descriptive by design and included no comparator group that operated without these modifications; it therefore cannot establish whether the modifications influence the complexity of subsequent transplantation, and no such inference should be drawn. Post-KPE portal hypertension and hepatopulmonary syndrome are markers associated with transplant requirement and should prompt intensified surveillance. The approach represents the standardisation of existing practice rather than a new technique.

## 1. Introduction

Kasai portoenterostomy (KPE) and liver transplantation after KPE have each been extensively studied, and the determinants of native liver survival are well characterised [1]. What has not been systematically examined is the operative link between them: whether the technical conduct of the index procedure—where the incision is placed, whether the liver is mobilised, how the Roux limb is configured—is chosen with the subsequent hepatectomy in view. KPE has been first-line therapy since its description in 1959, yet up to 70% of patients require transplantation within 20 years [2,3,4,5] KPE is therefore best understood not simply as a procedure to restore bile flow, but as the first operation in a two-stage strategy that culminates, in most patients, in hepatic replacement. Yet its technical conduct has been judged almost exclusively by native liver survival, with little attention to how operative decisions at KPE affect the safety and complexity of the transplantation that follows.

The two operations are anatomically linked: incision placement, the extent of liver mobilisation, and Roux limb configuration at KPE determine the adhesion burden, dissection planes, and bowel availability encountered at subsequent hepatectomy [6,7,8,9]. Whether these choices in fact translate into easier transplantation remains contested. A recent multicentre case-control study of 204 patients found that liver mobilisation at KPE—long avoided on the assumption that it increases adhesions—was associated with shorter operative times and lower transfusion requirements at subsequent transplantation, as well as with prolonged native liver survival [10,11]. None of these elements are new. By the early 2000s, KPE was already regarded in well-resourced centres performing both operations as the first stage of a two-stage strategy, and conducting the index procedure with the subsequent hepatectomy in view was accepted practice where a single team performs both [12,13,14]. Strategic incision placement, the preservation of ligamentous attachments, and the standardisation of Roux limb length have each been described individually [3,15,16,17,18,19,20]. This report therefore makes no claim of technical novelty. It describes the uniform application of these elements as a single protocol from the first case of an 11-year consecutive series and reports native liver survival and the transplant operative course in the resulting cohort.

We present an 11-year, single-surgeon experience of 74 consecutive KPE procedures at Schneider Children’s Medical Center, Israel. The primary aim was to describe a standardised transplant-orientated KPE approach applied uniformly across the series and to report native liver survival and the transplant operative course in the resulting cohort. Secondary aims were to identify post-KPE clinical events associated with eventual transplant requirement and characterise the transplant operative course in those who proceeded with transplantation. The study was designed as a descriptive consecutive series. Because no comparator group that operated without these modifications was available, it cannot be determined whether the modifications alter the complexity of subsequent transplantation, and no causal inference is intended.

## 2. Methods

### 2.1. Study Design and Population

This retrospective cohort study analysed prospectively maintained data on all patients who underwent KPE for BA at Schneider Children’s Medical Center, Israel, between January 2014 and September 2025. All subsequent liver transplantations were performed within the same institutional system, at the affiliated Department of Abdominal Organ Transplantation, Rabin Medical Centre–Beilinson Campus, by a shared paediatric hepatobiliary and transplant team performing 20–28 paediatric liver transplants annually throughout the study period. The protocol was approved by the Rabin Medical Center Institutional Review Board (0676-25-RMC—23 March 2026 up to 30 March 2027); consent was waived given the retrospective design.

AI Assistance: Grammarly (Grammarly Inc., San Francisco, CA, USA, desktop application v1.5, accessed August 2026) and Microsoft 365 Copilot (Microsoft Corporation, Washington, DC, USA, integrated in Microsoft Word) were used during manuscript preparation solely to improve language clarity, grammar, and readability. No generative AI tool was used to generate scientific content, perform data analysis, interpret results, or formulate conclusions. All scientific content, statistical analysis, and conclusions are the original work of the authors, who have reviewed and take full responsibility for the final text. 

**Figure 1:**
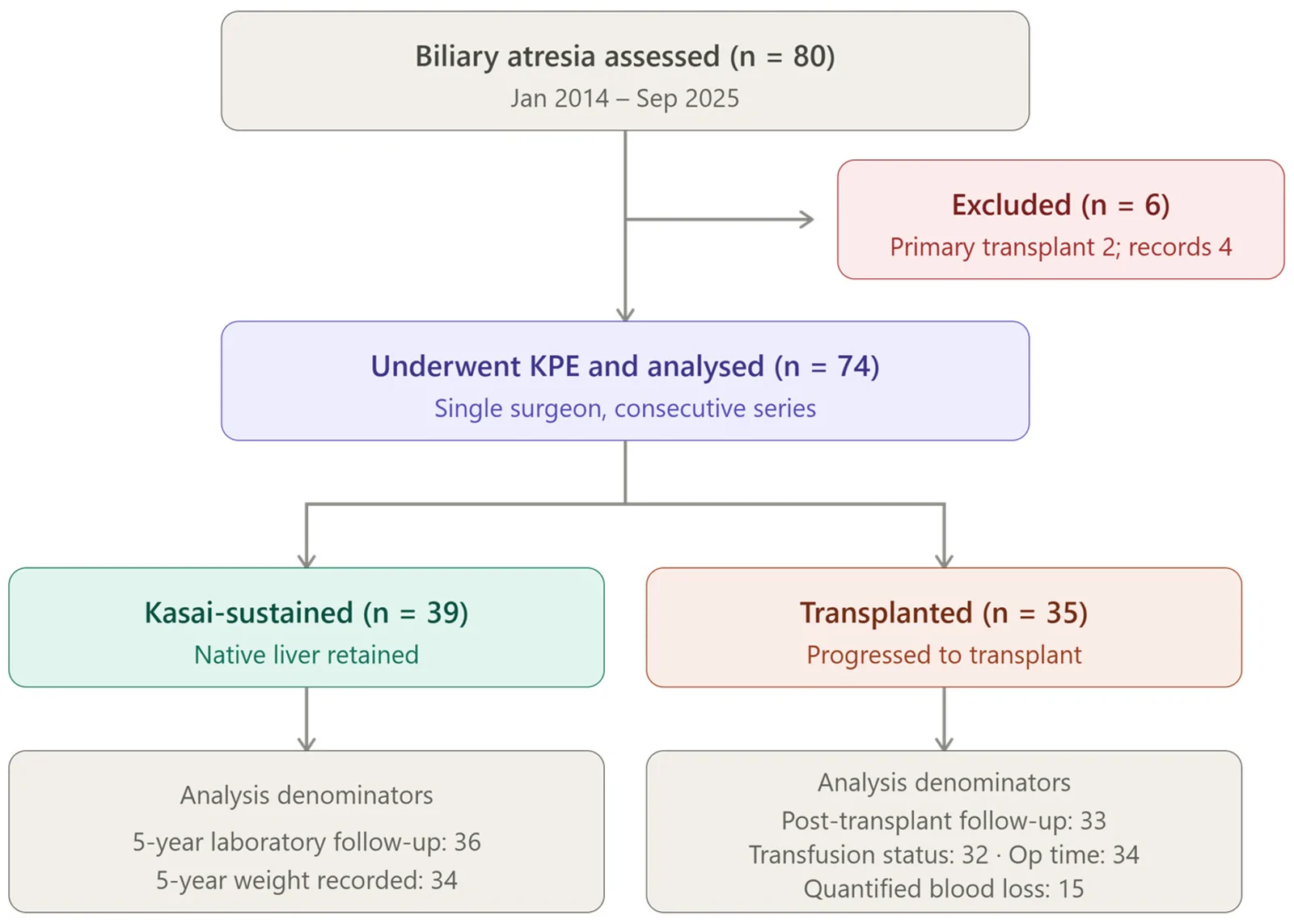
Patient inclusion. Of 80 patients with BA assessed at Schneider Children’s Medical Centre between January 2014 and September 2025, six were excluded: two who underwent primary liver transplantation without a prior KPE attempt and four with incomplete records. The remaining 74 consecutive patients underwent KPE and were analysed, comprising 39 who retained their native liver throughout follow-up and 35 who progressed to liver transplantation. Denominators for variables with incomplete documentation are shown for each group.

All consecutive patients diagnosed with BA who underwent KPE were included; those undergoing primary transplantation without a prior KPE attempt, or with incomplete records, were excluded (Figure 1). Diagnosis was confirmed by clinical presentation, laboratory findings, and hepatobiliary ultrasound, supported by liver biopsy and, where a patent gallbladder allowed, operative cholangiography demonstrating bile-duct occlusion. Patients were stratified by long-term outcome into a KPE-sustained group (native liver retained throughout follow-up) and a transplanted group.

### 2.2. Data Collection and Variables

Data were extracted from the prospectively maintained paediatric surgery and transplant databases and supplemented by electronic record review. Collected variables comprised sex, birth weight, associated anomalies (notably BA splenic malformation syndrome), age and weight at KPE, and pre-operative laboratory values (total bilirubin, albumin, ALT, AST, GGT, alkaline phosphatase, sodium, INR, creatinine). Disease severity was scored using the age-appropriate instrument— Paediatric End-Stage Liver Disease (PELD) for patients under 12 years and Model for End-Stage Liver Disease (MELD) for those 12 years and older—by the standard formula.

Post-KPE complications were systematically documented: failed KPE (no jaundice improvement 8 weeks post-KPE), failure to thrive (weight below the third percentile for age), portal hypertension (splenomegaly and/or varices on imaging, ascites, and thrombocytopenia), recurrent cholangitis (≥2 episodes requiring hospitalisation), gastrointestinal bleeding, liver synthetic dysfunction (coagulopathy, low albumin), and hepatopulmonary syndrome. Portal hypertension was defined clinically; portal pressure and hepatic venous pressure gradient were not measured. Hepatopulmonary syndrome was identified from the clinical diagnosis recorded in the electronic medical record; the underlying diagnostic investigations were not re-adjudicated retrospectively. Portopulmonary hypertension is a distinct entity characterised by elevated pulmonary artery pressure and was not systematically assessed; pulmonary artery pressure was not routinely recorded during the study period. Operative parameters for KPE included operative duration, estimated blood loss, and intraoperative complications. Operative parameters for transplantation included operative duration, estimated blood loss, whether blood loss exceeded 200 mL, and intraoperative red cell transfusion with volume where specified. Intraoperative complications at KPE were defined as any deviation from the expected operative course documented in the operative note, including measurable blood loss, transient haemodynamic instability requiring intervention, difficult exposure or adhesions, or inadvertent injury to adjacent structures. Events were recorded irrespective of severity or consequence, and the majority were minor; the variable therefore reflects intraoperative event documentation rather than clinically significant morbidity.

### 2.3. Surgical Technique and Modifications

All KPE procedures were performed by a single surgeon using a standardised transplant-orientated KPE approach, with three deliberate modifications applied uniformly from the first case. First, KPE was performed through a transverse bilateral subcostal incision 2 cm below the costal margin, positioned to align with future transplant access, thereby avoiding additional transplant incisions and confining adhesions to the perihepatic region rather than to bowel loops. Second, in contrast to the common practice of ligament detachment, mobilisation, and exteriorisation, the liver was kept in its anatomical position to limit adhesion formation between the cirrhotic liver, spleen, and retrohepatic inferior vena cava; all ligamentous attachments except the falciform were preserved since their division complicates subsequent hepatectomy. Third, a standardised 50 cm ascending Roux-en-Y jejunal limb was fashioned and transected proximally to the ligament of Treitz; the length was chosen to leave adequate bowel after partial resection of the portoenterostomy loop during hepatectomy. (Supplementary Figure S1).

A Rochard retractor with malleable plates for subhepatic visualisation; hilar dissection was performed under 6× loupe magnification with sharp excision of the fibrotic hilar plate. The Roux limb was anastomosed to the transected portal plate with interrupted 5-0 PDS sutures, and the entero–enterostomy was hand-sewn in two layers, positioned to avoid dissection planes relevant to future transplantation (Supplementary Figures S1 and S2).

### 2.4. Post-Operative Management and Follow-Up

All patients received standardised post-operative care: intravenous piperacillin–tazobactam for three days, ursodeoxycholic acid 20 mg/kg/day, and fat-soluble vitamin supplementation; cholangitis was treated with intravenous antibiotics followed by trimethoprim–sulfamethoxazole prophylaxis. Follow-up—clinical evaluation, growth parameters, and laboratory studies at each visit—was defined as the time from KPE to transplantation, death, or last documented contact, whichever came first. Precise per-patient follow-up dates were not consistently retrievable, and a reverse Kaplan–Meier median could not therefore be computed for the full cohort. Follow-up completeness is instead reported as the proportion of patients with recorded clinical and laboratory contact at pre-specified milestones. For transplanted patients, post-transplant follow-up was recorded per patient and is reported as a reverse Kaplan–Meier median.

Jaundice clearance was defined as total bilirubin < 2 mg/dL (34 μmol/L) within six months of KPE. Growth was tracked by serial weight and albumin at 1, 2, 3, 5, and 10 years. Short-term outcomes (within 30 days) included ICU and hospital stay, 30-day readmission, and 30-day mortality. For transplanted patients, additional outcomes were the KPE-to-transplant interval, listing indications, transplant operative time, blood loss, intraoperative red cell transfusion, and post-transplant survival at 1, 3, and 5 years, estimated by Kaplan–Meier.

### 2.5. Statistical Analysis

Analyses used R 4.3.0. Continuous variables are summarised as mean ± SD or median (IQR)—according to the Shapiro–Wilk test—and categorical variables as frequencies (%). Groups were compared with Student’s *t*-test or the Mann–Whitney U test for continuous variables, and the chi-square test or Fisher’s exact test was used for categorical variables. Predictors of transplantation were assessed by logistic regression (ever requiring transplant) and Cox proportional hazards regression (time to transplant); variables with univariate *p* < 0.10 entered multivariable models, with selection guided by clinical relevance and multicollinearity avoidance, and the proportional-hazards assumption was checked by Schoenfeld residuals. Predictors of KPE failure, defined as progression to liver transplantation, were assessed in a model containing age at KPE, pre-KPE total bilirubin, and GGT. This analysis was added during revision; covariates were fixed before fitting on the basis of established prognostic relevance, and no stepwise selection was applied. Firth penalised logistic regression with profile-likelihood confidence intervals was used given the number of events relative to covariates.

Post-KPE complications (portal hypertension, variceal bleeding, hepatopulmonary syndrome, recurrent cholangitis, gastrointestinal bleeding) were entered into the Cox model as fixed (ever/never) indicators because precise onset dates were not consistently retrievable across the study period. This does not fully capture the time-varying nature of these exposures and may overestimate hazard ratios through immortal-time bias; the estimates should therefore be read as associations between eventual complication status and time to transplantation rather than as time-dependent effects, and prospective recording of onset is recommended.

Transplant-free survival was estimated by Kaplan–Meier analysis for the overall cohort and stratified by sex, age at KPE (dichotomised at 60 days), and pre-operative complications, with groups compared using a log-rank test; patients alive with their native liver were censored at last follow-up. Statistical significance was two-sided at *p* < 0.05. Missing data were handled by complete-case analysis throughout; no values were imputed. The number of patients without a recorded value is reported for every variable in each table. Post-Kasai complications were not documented in all patients, and complication frequencies are reported over observed denominators rather than assuming absence where documentation was lacking; the multivariable models were fitted on the 60 patients with complete data for all model covariates (30 transplant events). The principal missingness affected transplant blood loss, quantified in 15 of 35 patients (42.9%); because an unrecorded value cannot be distinguished from a true reading of zero, the 20 undocumented patients were excluded from blood-loss summaries rather than imputed as zero, and the denominator (*n* = 15) is reported throughout. One transplant operative-time value was missing (*n* = 34). Missingness was likely not completely at random with respect to surgical era (recording practices evolved over the 11 years); sensitivity analyses excluding the affected variables did not alter the primary conclusions.

## 3. Results

### 3.1. Study Population and Demographics

**Figure 2:**
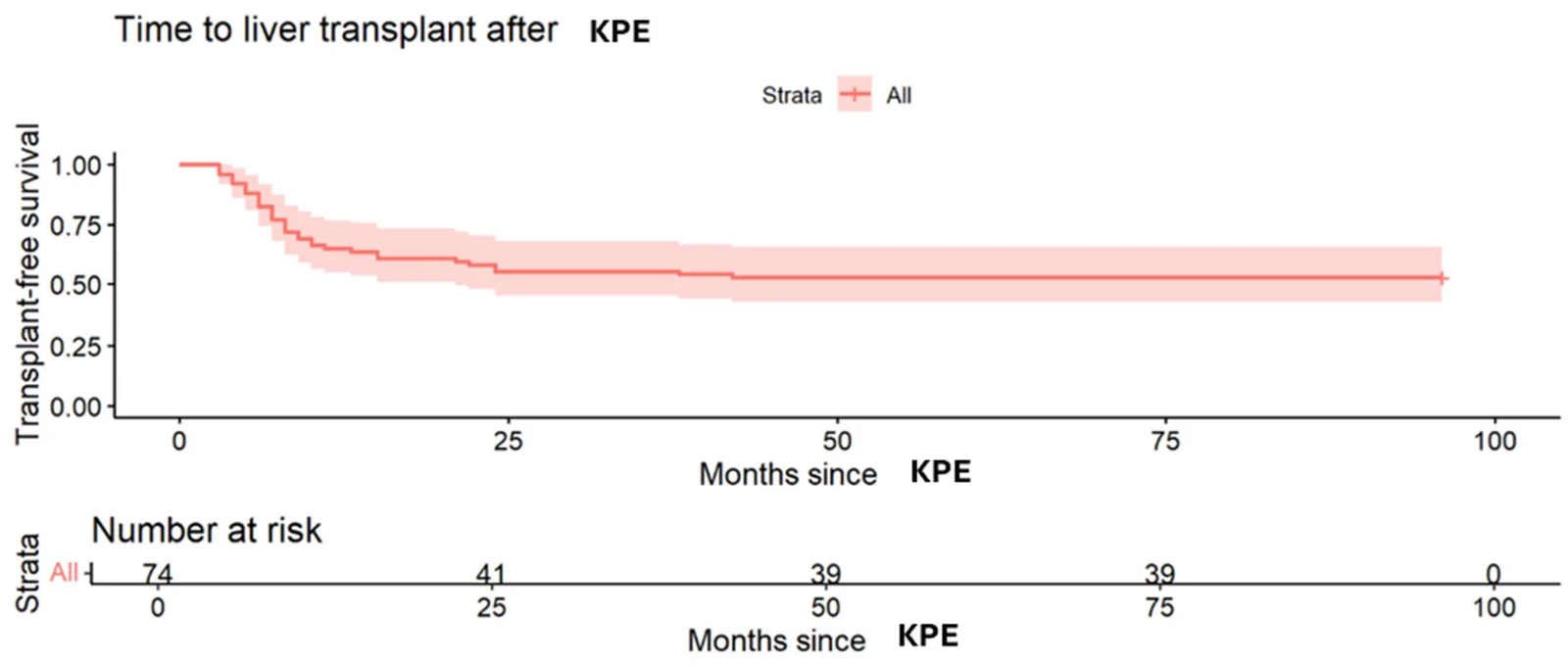
Overall transplant-free survival following KPE for the entire cohort (*n* = 74). The transplant-free survival rate at 50 months was approximately 53%, with the curve plateauing thereafter. The shaded area represents the 95% confidence interval. Numbers at risk are shown below the graph.

Patient inclusion is summarised in Figure 1. During the study period, 74 children with BA underwent KPE at our institution; 39 (52.7%) maintained their native liver (Kasai-sustained group) and 35 (47.3%) eventually required liver transplantation (transplanted group). A reverse Kaplan–Meier median follow-up could not be computed for the full cohort because per-patient follow-up dates were not consistently retrievable. Among the 39 Kasai-sustained patients, 36 (92.3%) had laboratory follow-up recorded at 5 years and 34 (87.2%) had recorded weight at 5 years; one was followed to 10 years. Among transplanted patients, median post-transplant follow-up estimated by reverse Kaplan–Meier was 5.0 years (range 1.0–10.0; *n* = 33). The Kaplan–Meier transplant-free survival curve plateaus at 50 months (Figure 2).

Table 1 presents baseline demographics. The cohort comprised 43.2% males, with no significant difference between groups (35.9% vs. 51.4% male, *p* = 0.266). The median gestational age was 39 (IQR 38.3–40.0) weeks and the median birth weight was 3.20 (2.90–3.60) kg. Graft type among transplanted patients is detailed in Table 2 and Table 3.

### 3.2. Pre-Kasai Clinical Profile

Pre-operative characteristics are detailed in Table 4. The median birth-to-KPE interval was 53 (IQR 44–60) days overall, with no significant difference between groups (Kasai-sustained 54 (45–69) days vs. transplanted 53 (42–59) days, *p* = 0.209). Weight at KPE was comparable (mean 4.36 ± 0.61 vs. 4.27 ± 0.72 kg, *p* = 0.578). Laboratory parameters were similarly non-discriminating: median total bilirubin of 8.30 (6.90–10.00) vs. 9.3 (7.5–11.8) mg/dL (*p* = 0.427) and median albumin of 3.90 (3.70–4.10) vs. 3.90 (3.65–4.20) g/dL (*p* = 0.341). No baseline parameter significantly distinguished patients who would eventually require transplantation.

### 3.3. Complications After KPE, by Eventual Outcome Group

All complications reported in this subsection were recorded after KPE and, in the transplanted group, before transplantation. Group labels denote eventual outcome and not status at the time of the event. Denominators differ between variables because documentation was incomplete for some events; the number of patients without a recorded value is given for each variable in Table 4.

Portal hypertension was documented in sixteen of thirty patients who subsequently required transplantation (53.3%) versus five of thirty-five who retained their native liver (14.3%; *p* = 0.001), as well as variceal bleeding in eleven of thirty-three (33.3%) versus two of thirty-one (6.5%; *p* = 0.012); these were the only between-group differences reaching statistical significance. Recurrent cholangitis occurred in 14 of 35 (40.0%) versus 10 of 39 (25.6%; *p* = 0.220). Hepatopulmonary syndrome occurred in three of thirty-three (9.1%) patients versus zero of thirty-two (0%; *p* = 0.238). Failure to thrive (6/35, 17.1% versus 8/39, 20.5%), ascites (2/35, 5.7% versus 2/39, 5.1%), and gastrointestinal bleeding (7/34, 20.6% versus 2/34, 5.9%) did not differ between groups.

Because group membership is defined by an event occurring after these complications were recorded, these are descriptions of the clinical course preceding transplantation; the corresponding time-to-event analyses are presented in Section 3.7.

### 3.4. KPE Procedure Outcomes

Jaundice clearance (total bilirubin < 2 mg/dL within six months of KPE) was achieved in 47/74 (63.5%). Table 2 and Table 3 detail operative and post-operative outcomes. In the Kasai-sustained group, median operative duration was 3.0 (IQR 2.5–4.5) hours; thirty-one of thirty-nine patients (79.5%) had no recorded blood loss and eight (20.5%) lost an estimated 50 mL. Intraoperative complications were documented in 13 patients (33.3%), with no intraoperative mortality. Median ICU stay was 2 (IQR 2–3) days and hospital stay was 12 [4,5,15,16,21,22,23,24] days; two patients had prolonged hospitalisation exceeding 100 days. The 30-day readmission rate was 17.9% (7/39), with no 30-day mortality.

**Figure 3:**
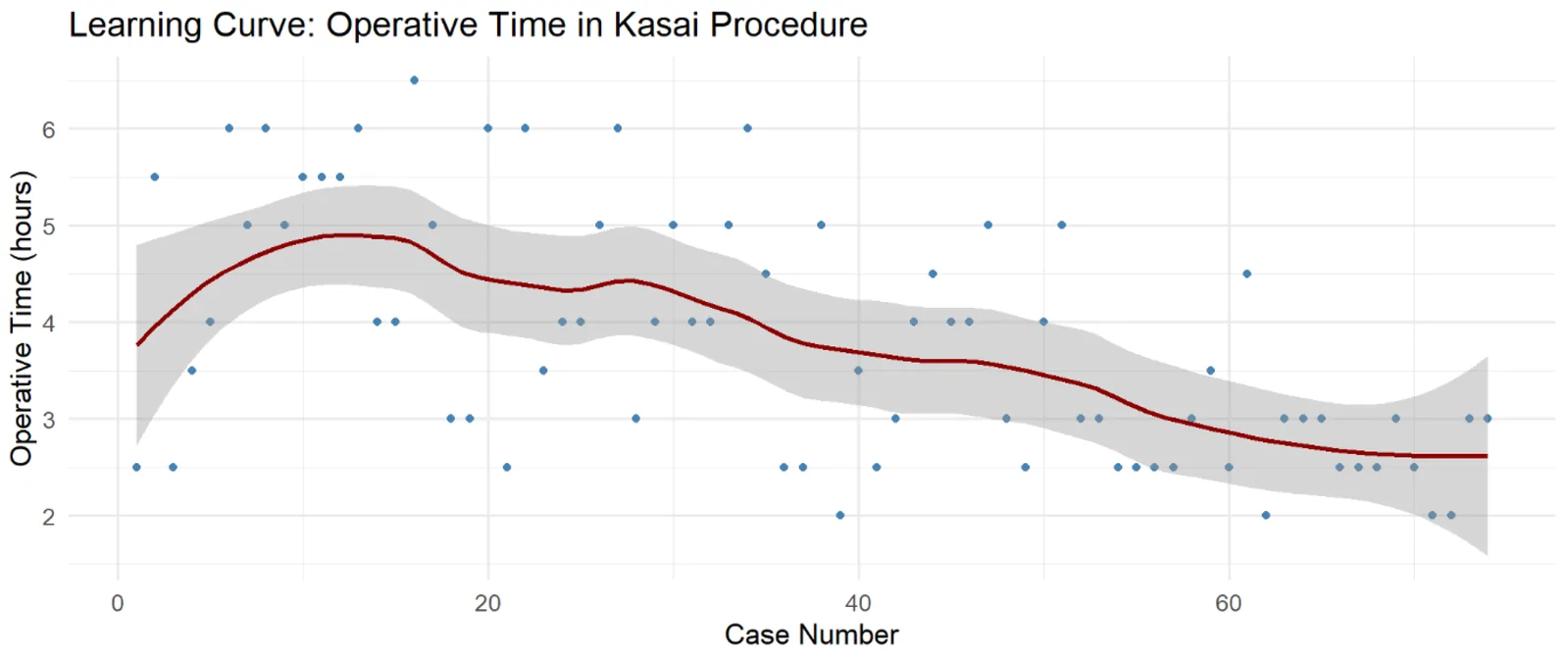
Operative time for KPE across an 11-year single-surgeon series. Individual cases are shown as points (*n* = 74), ordered chronologically by operation date, with a locally weighted smoothing (LOESS) regression line and 95% confidence interval. Operative duration declined across the series (Spearman ρ = −0.566, *p* < 0.001), with median duration falling from 5.0 h in the first sequence tertile to 4.0 and 3.0 h in the second and third tertiles. The three operative modifications described in the Methods were applied uniformly from the first case and did not change during the study period; consequently, technique and accumulating experience are perfectly confounded in this series, and the observed decline cannot be attributed to either. Changes in anaesthetic and peri-operative management over the study period were not controlled and may also contribute.

In patients who eventually required transplantation, the initial KPE showed a slightly longer median operative time of 4.0 (3.0–5.0) hours (*p* > 0.05), and intraoperative complications were more frequent (20/35, 57.1%). Post-operative course was comparable (median ICU stay 2 [13,25] days; hospital stay 11 [4,5,15,16,21,22,23,24] days), with a 30-day readmission rate of 34.3% (12/35). Across the cohort, operative duration decreased from approximately 4.8 h in early cases to 3.0 h after case 60 (Figure 3). Operative duration for KPE declined across the series. Median duration fell from 5.0 h in the first sequence tertile to 4.0 and then 3.0 h in the second and third tertiles (Spearman ρ = −0.566, *p* < 0.001; Figure 3). Transplant operative time declined in parallel with KPE case sequence (ρ = −0.586, *p* < 0.001, *n* = 34). Both groups showed progressive weight gain across follow-up; absolute weights at 1, 2, 3, 5, and 10 years are presented in Table 2 and Table 3.

### 3.5. Liver Transplantation Characteristics

Among the 35 transplanted patients (Table 3), the median time from KPE to transplantation was 8 (IQR 6–12) months and the median age at transplantation was 300 days (9.9 months) (IQR 225–495; range 150–1230). Listing followed standard paediatric criteria applied by the multidisciplinary team. The clinical events most frequently documented in the transplanted group before listing were portal hypertension (16/30, 53.3%), recurrent cholangitis (14/35, 40.0%), variceal bleeding (11/33, 33.3%), and failed KPE with persistent jaundice (Table 4). These are complication frequencies rather than a structured audit of listing indications, which were not recorded in a dedicated field. Twenty-six patients (74.3%) received a left lateral segmental graft (Segments 2–3) and nine (25.7%) received a full liver graft.

The median transplant operative time was 8.0 (IQR 6.0–10.2) hours (*n* = 34). Intraoperative transfusion status was documented in 32 of 35 transplanted patients (91.4%). Fourteen required intraoperative red cell transfusion (43.8% of those documented): one unit in six patients, two units in four, three units in three, and an unspecified volume in one. Fourteen patients had no intraoperative event recorded, and four had another event documented (two unplanned reoperations and two requiring vasoactive support). Intraoperative blood loss exceeding 200 mL was recorded in 12 of 35 patients (34.3%); this threshold was documented for all transplanted patients, whereas quantified volumes were available in 15 (median 300 mL (IQR 205–450)), of whom 11 exceeded 200 mL. No 30-day transplant mortality occurred.

### 3.6. Survival Outcomes

**Figure 4:**
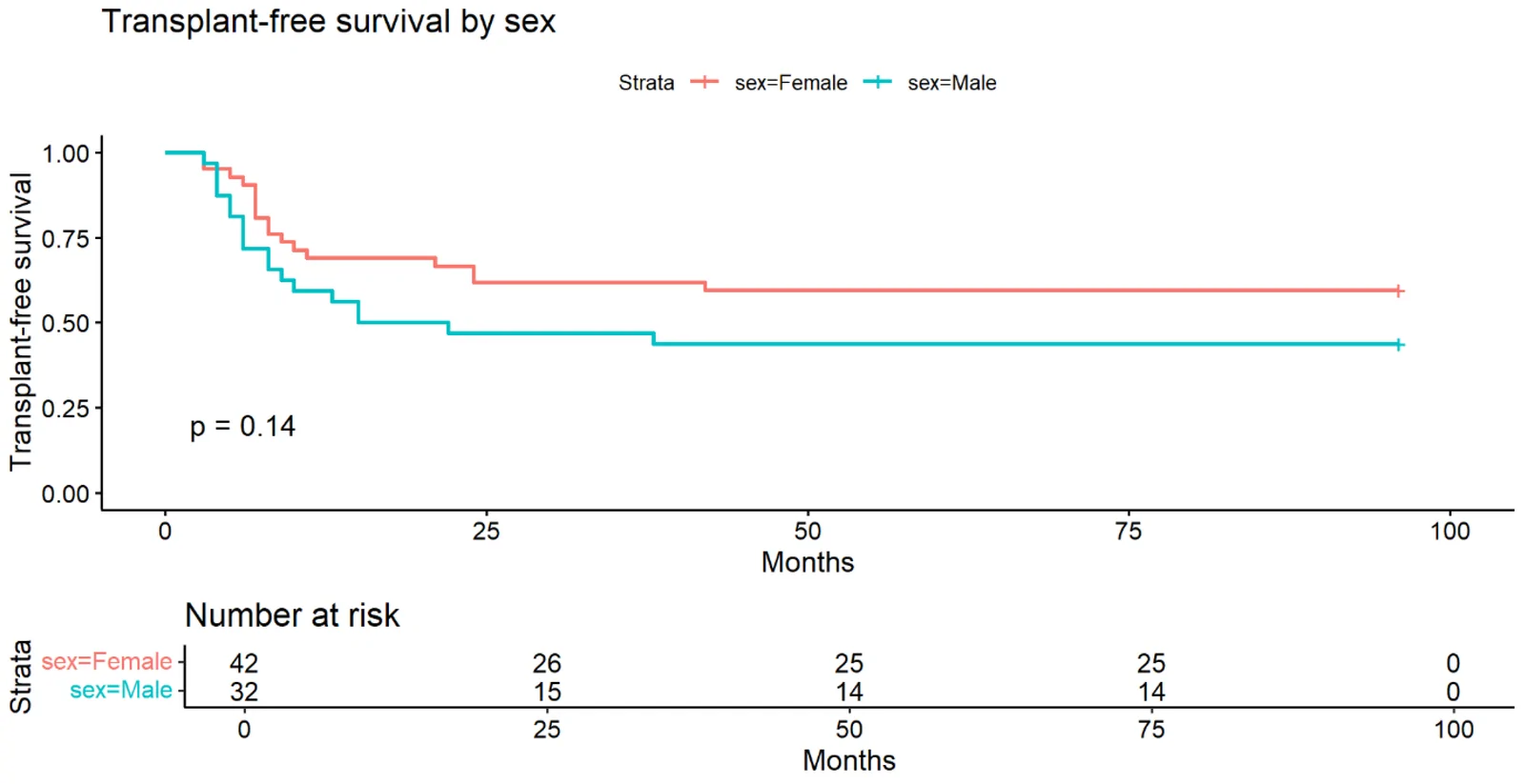
Kaplan–Meier curves showing transplant-free survival after KPE stratified by sex. Female patients (red line) demonstrated a trend toward improved transplant-free survival compared to males (blue line), though this difference was not statistically significant (log-rank *p* = 0.14). Numbers at risk are shown below the graph.

Table 5 summarises survival. There was no intraoperative or 30-day mortality after KPE. One death occurred in the cohort, 2.0 years after liver transplantation and 3.8 years after KPE, yielding an overall survival of 73 of 74 patients (98.6%). Post-transplant Kaplan–Meier survival was 100% at one year (*n* = 33 at risk), 96.4% at three years (*n* = 23), and 96.4% at five years (*n* = 16) based on the 33 transplanted patients with recorded post-transplant follow-up (Table 3). Transplant-free survival at 50 months was approximately 53% (Figure 2). Stratified by sex, transplant-free survival did not differ significantly (log-rank *p* = 0.14), although females showed a numerically but not statistically higher rate (approximately 60% vs. 45% at 50 months; Figure 4).

### 3.7. Predictors of KPE Failure

In the pre-specified model, no baseline variable was associated with progression to transplantation: age at KPE (OR 0.85 per 10 days, 95% CI 0.66–1.07, *p* = 0.178), pre-KPE total bilirubin (OR 1.06 per mg/dL, 95% CI 0.93–1.21, *p* = 0.376), and GGT (OR 1.01 per 100 U/L, 95% CI 0.93–1.11, *p* = 0.783). Univariable comparisons were concordant (Table 4).

### 3.8. Predictors of Transplantation

**Figure 5:**
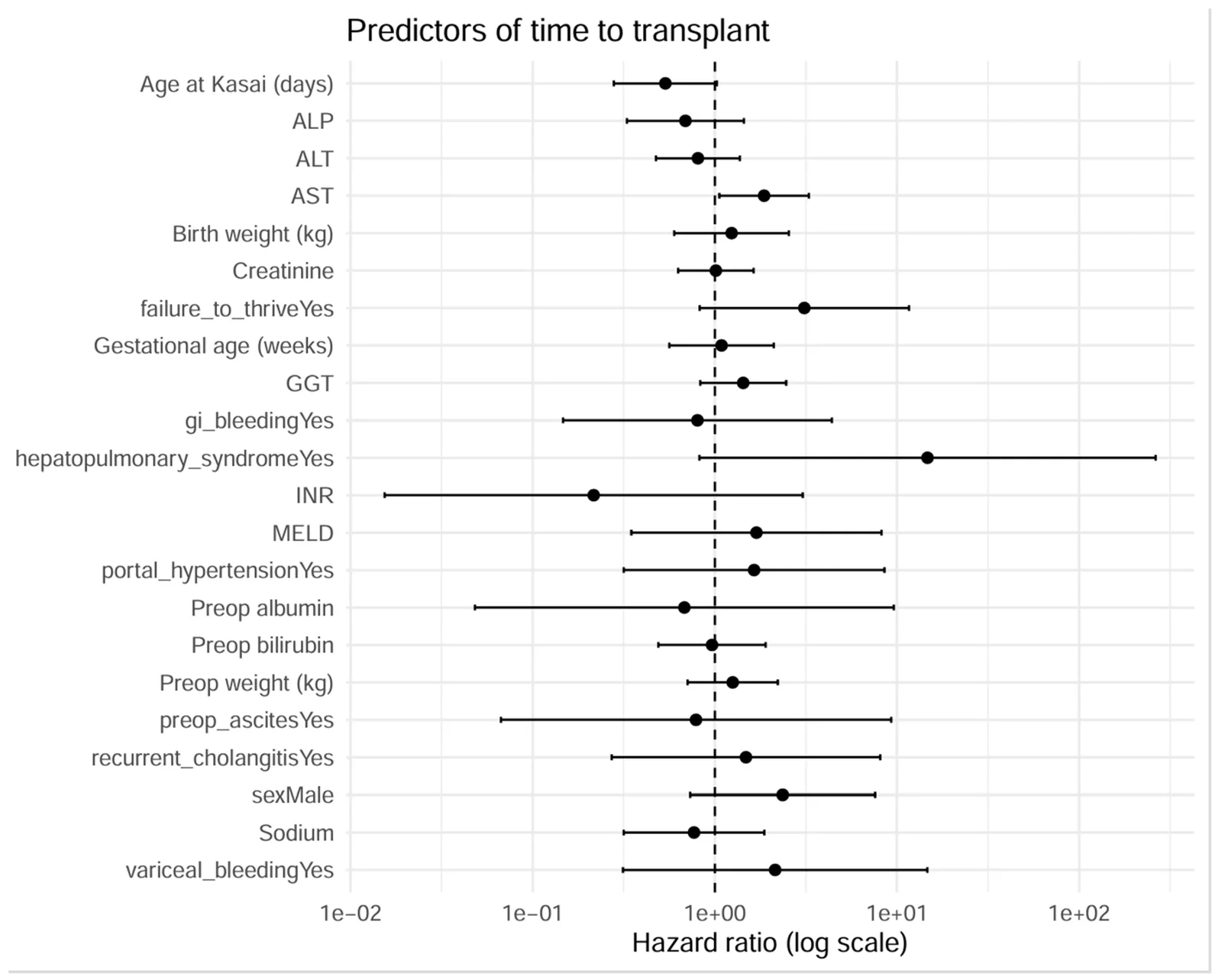
Forest plot of univariate Cox regression analysis for predictors of time to liver transplantation following KPE. Hazard ratios (HR) with 95% confidence intervals are displayed on a logarithmic scale. Variables are ordered alphabetically. The vertical dashed line at HR = 1.0 represents no effect. Hepatopulmonary syndrome demonstrated the strongest association with earlier transplantation (HR ~10), followed by portal hypertension and variceal bleeding. Variables with confidence intervals crossing 1.0 showed no significant association with time to transplantation in a univariate analysis. These results were used to select candidates (*p* < 0.10) for the multivariate Cox model, which ultimately identified portal hypertension (aHR 3.63, *p* = 0.008) and hepatopulmonary syndrome (aHR 10.17, *p* = 0.003) as independent predictors.

Table 6A,B presents predictors of eventual transplantation. In the multivariable Cox proportional-hazards model, post-KPE portal hypertension (adjusted HR 3.63, 95% CI 1.40–9.42, *p* = 0.008) and hepatopulmonary syndrome (adjusted HR 10.23, 95% CI 2.19–47.85, *p* = 0.003) were independent predictors of time to transplantation; the hepatopulmonary syndrome estimate is based on only three patients identified from recorded clinical diagnoses that were not re-adjudicated—and in whom portopulmonary hypertension could not be excluded—and must be interpreted with caution. In the univariate Cox analysis, portal hypertension (HR 3.11, 95% CI 1.51–6.40, *p* = 0.002), hepatopulmonary syndrome (HR 5.13, 95% CI 1.48–17.71, *p* = 0.010), and variceal bleeding (HR 2.48, 95% CI 1.19–5.15, *p* = 0.015) were each associated with time to transplantation (Figure 5).

Logistic regression for the binary outcome of ever requiring transplantation yielded concordant results: portal hypertension was the strongest predictor (univariate OR 6.86, 95% CI 2.21–24.58, *p* = 0.001; adjusted OR 7.41, 95% CI 1.51–55.42, *p* = 0.023), followed by variceal bleeding (OR 7.25, 95% CI 1.72–50.04) and gastrointestinal bleeding (OR 4.15, 95% CI 0.91–29.44). Hepatopulmonary syndrome was not estimable by this method owing to complete separation, as all three affected patients required transplantation (Table 6A,B).

## 4. Discussion

### 4.1. Principal Findings

In this single-surgeon series of 74 consecutive KPE procedures performed with a standardised transplant-orientated approach, 52.7% of patients retained their native liver; the operative course of those who required transplantation is described in Table 3. The approach codifies practices that have already been established in centres performing both operations, and its individual components have been reported previously [12,13,14,15,16,17,18,19,20]; the contribution of this series is the uniform application of a protocol across a consecutive cohort and the description of the resulting outcomes. Post-KPE portal hypertension and hepatopulmonary syndrome were the events most strongly associated with eventual transplantation. Both groups gained weight across follow-up, though without weight-for-age z-scores, these trajectories cannot be read as confirming age-appropriate growth. These findings support the continued role of portoenterostomy as first-line therapy for BA and, given that most patients ultimately require hepatic replacement, the value of conducting KPE with the subsequent transplant in view.

### 4.2. Comparison with the Previous Literature

Our native liver survival of 52.7% lies in the upper range of reported series, which vary from 31% at 10 years in the Taiwan cohort [22], 41% in the King’s College series [26], and 43–47% in the French and Canadian experiences [21,27], to 70.7% at 10 years in the Hong Kong territory-wide study [23]. The Japanese registry reported 54% at 5 years, falling to 45.5% at 10 years [28]. This variation reflects differences in patient selection, referral patterns, operative timing, surgical volume, and post-operative management. Notably, our median age at a KPE of 53 days matches the suboptimal timing reported nationally in the United States (57 days) [16], indicating that our outcomes reflect factors beyond timing alone. That native liver survival represents a spectrum rather than a cure is underscored by the Birmingham series, in which only 30% of patients reaching adult care retained their native liver, most with chronic liver disease—reinforcing the need for lifelong surveillance even in apparent KPE successes.

Prior KPE does not compromise transplant outcomes and may improve pre-transplant status by allowing children to reach a size and condition compatible with safer grafting [24,29]. In the present cohort, median transplant operative time was 8.0 h (IQR 6.0–10.2) (*n* = 34), intraoperative blood loss was documented in 15 of 35 patients (median 300 mL (IQR 205–450)), and bowel injury during adhesiolysis occurred in one of thirty-two patients (3.1%). We do not compare these values with previously published series. The available literature differs substantially in era, donor type, living versus deceased grafting, recipient age, centre experience, immunosuppression, and operative technique, and no systematic search was undertaken to identify comparable cohorts; any numerical contrast would therefore be uninterpretable. These parameters are reported to characterise the operative course of the present series, not to benchmark it.

### 4.3. Possible Mechanisms

This study provides no direct mechanistic evidence linking the operative modifications to the observed outcomes, and the following rationale is therefore hypothetical. Aligning the transverse subcostal incision with standard transplant access may reduce the adhesiolysis required when incision planes intersect [30,31]. Keeping the liver in situ may preserve tissue planes between the liver capsule, diaphragm, retrohepatic inferior vena cava, and spleen that exteriorisation would otherwise disrupt. Standardising the Roux limb at 50 cm is intended to leave adequate bowel after partial resection of the portoenterostomy loop during hepatectomy. Crucially, the principal determinants of a difficult explant—portal hypertension with portal venous sclerosis or thrombosis and extensive collateral varices—are vascular and inflammatory rather than adhesive, and are not addressed by these modifications; consistent with this, post-KPE portal hypertension rather than adhesion burden was the dominant predictor of transplant requirement in our cohort. The modifications therefore target a narrower pathway—adhesion- and bowel-related injury during adhesiolysis—which this series was not designed to measure with any validated instrument. Because transplant operative time and blood loss are multifactorial, reflecting recipient severity, graft quality, intraoperative management, team experience, and era, the independent contribution of the index KPE cannot be isolated in this design; that baseline severity markers did not differ between groups (Table 4) argues against the selection of a less ill recipient population but does not exclude residual confounding. The evidence bearing on the second of our three modifications does not support our rationale. A multicentre retrospective comparison of 142 patients undergoing KPE with liver mobilisation against 62 without found that at subsequent transplantation, the mobilised group had shorter operative times (295 versus 510 min) and lower transfusion volumes (55 versus 96 mL/kg), alongside prolonged native liver survival; mobilisation was associated with greater intraoperative haemodynamic compromise and transient post-operative hepatic stress that resolved within seven days [11]. A smaller comparative study of liver exteriorisation reached broadly concordant peri-operative conclusions [32,33], and adhesion-sparing eversion techniques have been proposed as an intermediate approach [34]. Our series, which contains no non-mobilised comparator, cannot adjudicate this question. We therefore present the avoidance of mobilisation as the practice adopted in this series rather than as an evidence-based recommendation and note that the only controlled evidence currently available points in the opposite direction.

The confounding note above is structural rather than incidental: with the protocol applied uniformly from the first case, technique and accumulating experience covary perfectly across the series, and transplant operative time declines in parallel with the KPE case sequence (ρ = −0.586, *p* < 0.001), so any transplant-side improvement is as readily explained by eleven years of accumulating surgical, anaesthetic, and intensive-care experience as by the modifications themselves.

Portal hypertension was documented in sixteen of thirty patients who subsequently required transplantation (53.3%) versus five of thirty-five who retained their native liver (14.3%), reflecting the progressive hepatic fibrosis and sinusoidal obstruction that ultimately overcome the bile drainage achieved at KPE [23]. Recurrent cholangitis was more frequent in transplanted patients (40.0% vs. 25.6%) with a non-significant univariate association (HR 1.63) and did not qualify for the multivariable model, and per-patient temporal sequencing was not retrievable. The proposed pathway is nonetheless biologically coherent: recurrent ascending cholangitis accelerates fibrosis, which drives portal hypertension and, in advanced disease, the pulmonary vascular complications of cirrhosis [35,36]—positioning cholangitis as an upstream initiator and our identified predictors as late expressions of the same fibro-obliterative trajectory. Prospective documentation of cholangitis episodes with parallel surveillance would allow for this sequence to be tested directly.

### 4.4. Clinical Implications

No baseline variable predicted KPE failure in this cohort. The null result for age at KPE most likely reflects restricted range—the interquartile range was 43.5 to 60 days—rather than absence of an association, which is well established across wider age distributions [18]. Post-KPE portal hypertension (adjusted HR 3.63) and hepatopulmonary syndrome (adjusted HR 10.23) were the strongest markers of eventual transplantation, though both were modelled as fixed covariates and the hepatopulmonary syndrome estimate rests on three patients (see Limitations). Clinically, both are better read as markers of transplant need than as automatic indications. Portal hypertension emerging post-KPE should prompt intensified surveillance—regular clinical and Doppler assessment, endoscopic screening where jaundice persists, and early listing evaluation—rather than immediate listing; patients without it can follow standard surveillance, with elastography from age five. Systematic pulse-oximetry and contrast-echocardiography screening may identify hepatopulmonary syndrome before severe hypoxaemia develops. More broadly, a dichotomous success/failure categorisation understates the procedure’s value: patients who ultimately required transplantation retained native liver function through a critical period of growth and 52.7% avoided transplantation altogether. This addresses the adhesion, malnutrition, and delayed-therapy concerns raised by advocates of primary transplantation [3]; the 2024 meta-analysis of 3276 patients found no adverse effect of prior KPE on transplant outcomes [24], and cost-effectiveness modelling favours a KPE-first strategy [5]. Priorities for prospective work centre on the direct measurement of operative difficulty at transplantation, which this series could not provide. A prospective protocol should record a validated adhesion score, a composite transplant difficulty score, the isolated hepatectomy time, hilar dissection time, graded portal venous and caval dissection difficulty, and the need for and type of vascular reconstruction as well as systematic echocardiographic assessment with estimation of pulmonary artery pressure, permitting the formal distinction of portopulmonary hypertension from hepatopulmonary syndrome. Moreover, the prospective grading of unexpected intraoperative events should be performed using the validated Clavien–Madadi classification for paediatric surgery [30,37]. Further priorities include multicentre validation with a non-modified comparator arm, video-based standardisation of the operative protocol, integration with the infant BA Liver Fibrosis score [9] and elastography, and the adoption of weight-for-age z-scores and patient-reported outcomes in place of absolute weight trajectories [15,22,38,39,40,41].

### 4.5. Limitations

Four constraints qualify these findings.

Design. The series is retrospective, single-centre, and single-surgeon. Because the modifications were applied uniformly from the first case, no unmodified comparator exists within the series, and the technique is perfectly confounded with accumulating experience: transplant operative time declined in parallel with KPE case sequence (ρ = −0.586, *p* < 0.001), and published learning curves for KPE exceed 50 cases [37]. The 11-year span also encompasses uncontrolled changes in immunosuppression, equipment, and team composition. Multicentre or registry-based comparison is the appropriate confirmatory step.

Measurement. Operative difficulty at transplantation was not measured. No validated adhesion score or composite difficulty index was applied, the hepatectomy and hilar dissection phases were not timed separately, portal venous and caval dissection difficulty were not graded, and vascular reconstruction was not systematically recorded. Operative time, transfusion requirement and bowel injury describe the operative course but do not quantify its difficulty. Portal hypertension was defined clinically without pressure measurement; pulmonary artery pressure was not recorded, so portopulmonary hypertension could not be excluded in the three patients with hepatopulmonary syndrome. Growth is reported as absolute weight, weight-for-age z-scores being unavailable.

Missing data. Quantified transplant blood loss was available in 15 of 35 patients (42.9%) and is reported over the observed denominator rather than imputed; the dichotomous threshold of >200 mL was documented for all. Per-patient follow-up dates could not be reconstructed, precluding a cohort-wide reverse Kaplan–Meier median. Interval bilirubin, fibrosis staging, CMV serology, steroid exposure, and BA splenic malformation status were not uniformly retrievable, so predictors of jaundice clearance could not be examined.

Analysis. Post-KPE complications entered the Cox model as fixed (ever/never) covariates because onset dates were unavailable; this is susceptible to immortal-time bias and probably inflates the reported hazard ratios. The small number of events produced wide logistic-regression intervals (adjusted OR 7.41, 95% CI 1.51–55.42), and the Cox model is therefore treated as the primary model.

Consequently, this series cannot support any inference that the described modifications facilitate subsequent transplantation.

## 5. Conclusions

In an 11-year single-surgeon series, a standardised transplant-orientated KPE approach—a codification of practice already established in units performing both KPE and transplantation rather than a new technique—was applied uniformly to 74 consecutive patients, of whom 52.7% maintained their native liver. Transplantation in the remainder was performed without peri-operative mortality. Post-KPE portal hypertension and hepatopulmonary syndrome were associated with transplant requirement and may warrant intensified surveillance. This series was descriptive by design and included no comparator group that operated without these modifications; whether the transplant-orientated modification of KPE affects the complexity of subsequent transplantation remains untested and requires a controlled, prospective, multicentre design.

## Figures and Tables

**Table 1:** Demographics and baseline characteristics.

Variable	Overall (***n*** = 74)	Kasai-Sustained (***n*** = 39)	Transplanted (***n*** = 35)	***p***-Value
Sex, n (%)				0.266
Male	32 (43.2)	14 (35.9)	18 (51.4)	
Female	42 (56.8)	25 (64.1)	17 (48.6)	
Gestational age (weeks), median (IQR)	39 (38.3–40.0)	39 (38.0–40.0)	39 (38.4–40.0)	0.875
Birth weight (kg), median (IQR)	3.20 (2.90–3.60)	—	—	0.176

Data presented as median (IQR) or *n* (%).

**Table 2:** Outcomes following KPE and subsequent liver transplantation.

Variable	Kasai-Sustained (***n*** = 39)	Transplanted (***n*** = 35)	***p***-Value
Panel 1—KPE operative parameters and post-operative course			
KPE operative duration (hours), median (IQR)	3.0 (2.5–4.5)	4.0 (3.0–5.0)	NS
KPE blood loss > 0 mL, n (%) (all 50 mL)	8 (20.5)	5 (14.3)	NS
Intraoperative complications at KPE, n (%)	13 (33.3)	20 (57.1)	0.039
Intraoperative mortality at KPE, n (%)	0 (0.0)	0 (0.0)	—
ICU stay after KPE (days), median (IQR)	2 (2,3)	2 (2,3)	NS
Hospital stay after KPE (days), median (IQR)	12 (9–16)	11 (9–16)	NS
30-day readmission after KPE, n (%)	7 (17.9)	12 (34.3)	NS
30-day mortality after KPE, n (%)	0 (0.0)	0 (0.0)	—
Panel 2—Long-term anthropometric and synthetic-function parameters			
Weight, pre-operative (kg), mean ± SD	4.36 ± 0.61	4.27 ± 0.72	0.578
Weight at 1 year (kg), mean ± SD	8.61 ± 1.75	8.95 ± 1.52	NS
Weight at 2 years (kg)	12.0 (11.3–12.5) *	12.41 ± 1.81 †	NS
Weight at 3 years (kg)	15.0 (15.0–15.0) *	15.13 ± 1.72 †	NS
Weight at 5 years (kg)	19.0 (19.0–19.0) * (*n* = 34)	18.0 (16.9–20.4) * (*n* = 35)	NS
Weight at 10 years (kg)	28.7 (*n* = 1)	34.0 and 38.0 (*n* = 2)	—
Albumin, pre-operative (g/dL), median (IQR)	3.90 (3.70–4.10)	3.90 (3.65–4.20)	0.341
Albumin at 1 year (g/dL), mean ± SD	3.98 ± 0.47	3.76 ± 0.71	NS
Albumin at 2 years (g/dL), median (IQR)	4.00 (4.00–4.30)	4.20 (3.90–4.45)	NS
Albumin at 3 years (g/dL), median (IQR)	4.00 (4.00–4.15)	4.10 (4.00–4.50)	NS
Albumin at 5 years (g/dL), median (IQR)	4.00 (4.00–4.15) (*n* = 36)	4.20 (4.20–4.40) (*n* = 35)	NS
Albumin at 10 years (g/dL)	4.55 ± 0.07 (*n* = 2)	4.20 ± 0.49 (*n* = 4)	—

* Median (IQR) for non-normally distributed variables. † Mean ± SD where the subgroup distribution passed Shapiro–Wilk normality testing. Intraoperative complications at KPE were defined as any documented deviation from the expected operative course, irrespective of severity; see Methods. The variable reflects intraoperative event documentation rather than clinically significant morbidity. NS = not significant (*p* > 0.05). Statistical tests as specified in Methods. Documented events were more frequent in patients who subsequently required transplantation (57.1% vs. 33.3%, *p* = 0.039); given the low specificity of this variable, this difference is unlikely to reflect a meaningful difference in operative difficulty. Footnote: Two patients in the Kasai-sustained group required prolonged hospitalisation > 100 days due to post-operative complications.

**Table 3:** Liver transplantation parameters and post-transplant outcomes (transplanted patients, *n* = 35).

Variable	Value
Time from KPE to transplantation (months), median (IQR)	8 (6–12)
Transplant operative time (hours), median (IQR)	8.0 (6.0–10.2) (*n* = 34) ‡
Patients with quantified intraoperative blood loss, *n* (%)	15/35 (42.9)
Blood loss among quantified cases (mL), median (IQR)	300 (205–450)
Blood loss > 200 mL, *n* (%)	12/35 (34.3)
Blood loss > 200 mL among quantified cases, *n* (%)	11/15 (73.3)
Intraoperative red cell transfusion, *n* (%) §	14/32 (43.8)
Transfusion volume—1 unit, n	6
Transfusion volume—2 units, n	4
Transfusion volume—3 units, n	3
Transfusion volume—unspecified, n	1
Other intraoperative event, *n* (%) §	4/32 (12.5)
Graft type—full liver, *n* (%)	9 (25.7)
Graft type—left lateral segment (segments 2–3), *n* (%)	26 (74.3)
Transplant 30-day mortality, *n* (%)	0 (0.0)
Post-transplant follow-up (years), median (range) ‖	5.0 (1.0–10.0) (*n* = 33)
Post-transplant 1-year survival, % (*n* at risk) ‖	100 (33)
Post-transplant 3-year survival, % (*n* at risk) ‖	96.4 (23)
Post-transplant 5-year survival, % (*n* at risk) ‖	96.4 (16)

‡ One transplant operative record missing. § Intraoperative transfusion status was documented in 32 of 35 transplanted patients; three operative records lacked sufficient detail. “Other intraoperative event” comprised two unplanned reoperations and two patients requiring vasoactive support. This field recorded transfusion and related intraoperative events and is not comparable with the intraoperative complication variable reported for KPE in Table 2. ‖ Post-transplant survival estimates are Kaplan–Meier, based on the 33 transplanted patients with post-transplant follow-up recorded; one death occurred 2.0 years after transplantation. Median post-transplant follow-up was estimated by reverse Kaplan–Meier. Footnote: Quantified blood-loss volumes are restricted to the 15 of 35 patients with values documented in the operative note; the remaining 20 had no quantified value recorded and were excluded from volume-based summaries rather than imputed as zero since true zero loss cannot be retrospectively distinguished from absent documentation. The dichotomous threshold of blood loss exceeding 200 mL was documented separately for all 35 patients and is therefore reported without missingness. Graft type assignment for four patients with missing operative records was based on clinical review (three, full liver; one, segmental).

**Table 4:** Pre-KPE clinical profile and post-KPE complications.

Variable	Overall (***n*** = 74)	Kasai-Sustained (***n*** = 39)	Transplanted (***n*** = 35)	Missing, n	***p***-Value
Clinical Parameters					
Birth-to-Kasai interval (days) *	53 (44–60)	54 (45–69)	52.8 ± 12.4 †	0	0.209
Weight at KPE (kg), mean ± SD	4.32 ± 0.66	4.36 ± 0.61	4.27 ± 0.72	0	0.578
Laboratory Values					
Total bilirubin (mg/dL) *	8.40 (7.03–10.88)	8.30 (6.90–10.00)	9.78 ± 3.04 †	0	0.427
Albumin (g/dL) *‡	3.90 (3.68–4.20)	3.90 (3.70–4.10)	3.90 (3.65–4.20)	1	0.341
ALT (U/L) *	152 (95–203)	153 (120–207)	152 (91–194)	0	0.722
AST (U/L) *	214 (160–290)	212 (160–281)	217 (163–319)	1	0.355
GGT (U/L) *	478 (291–777)	486 (285–748)	450 (318–792)	0	0.747
ALP (U/L) *	550 (413–689)	550 (414–687)	554 (417–680)	0	0.656
Sodium (mmol/L) *	136 (134–137)	136 (135–138)	135.4 ± 2.2 †	0	0.125
INR *	0.97 (0.93–1.05)	0.99 (0.93–1.06)	0.95 (0.92–1.02)	0	0.324
Creatinine (mg/dL) *	0.21 (0.18–0.25)	—	—	0	0.806
MELD score *	16 (15–18)	16 (14–17)	17.0 ± 2.4 †	0	0.402
Post-KPE complications during follow-up, *n* (%)					
Failure to thrive	14 (18.9)	8 (20.5)	6 (17.1)	0	0.773
Ascites	4 (5.4)	2 (5.1)	2 (5.7)	0	1.000
Recurrent cholangitis	24 (32.4)	10 (25.6)	14 (40.0)	0	0.220
Portal hypertension	21/65 (32.3)	5/35 (14.3)	16/30 (53.3)	9	0.001
Gastrointestinal bleeding	9/68 (13.2)	2/34 (5.9)	7/34 (20.6)	6	0.150
Hepatopulmonary syndrome	3/65 (4.6)	0/32 (0.0)	3/33 (9.1)	9	0.238
Variceal bleeding	13/64 (20.3)	2/31 (6.5)	11/33 (33.3)	10	0.012

* Median (IQR) (Shapiro–Wilk *p* < 0.05 for overall distribution). † Mean ± SD where subgroup passed normality (SW *p* > 0.05). ‡ Patient #50 excluded (entry 210.0 g/dL: data entry error under verification). ALT: alanine aminotransferase; AST: aspartate aminotransferase; GGT: gamma-glutamyl transferase; ALP: alkaline phosphatase; INR: international normalised ratio; MELD: Model for End-Stage Liver Disease. Complication frequencies are reported over observed denominators; patients without documentation were excluded rather than assumed to be event-free. Missingness was concentrated in a small number of patients with sparse follow-up records: sixty-one of seventy-four patients had all four late complications documented, while five patients had none documented.

**Table 5:** Post-KPE survival outcomes (overall cohort, *n* = 74).

Outcome	n (%)
Intraoperative mortality	0 (0.0)
30-day mortality	0 (0.0)
1-year survival	74 (100.0)
3-year survival	74 (100.0)
5-year survival	73 (98.6)
Overall survival, *n* (%)	73/74 (98.6)

One death occurred in the cohort, 2.0 years after liver transplantation and 3.8 years after KPE. Survival at fixed intervals from KPE could not be estimated for the full cohort, as exact follow-up dates were not retrievable for patients retaining their native liver; 36 of 39 (92.3%) had documented laboratory follow-up at five years. Post-transplant Kaplan–Meier survival is reported in Table 3.

**Table 6:** (A) Univariate analysis: predictors of liver transplantation. (B) Multivariate analysis: independent predictors of liver transplantation.

(A)				
Variable	Logistic OR (95% CI)	*p*-value	Cox HR (95% CI)	*p*-value
Portal hypertension	6.86 (2.21–24.58)	0.001	3.11 (1.51–6.40)	0.002
Variceal bleeding	7.25 (1.72–50.04)	0.016	2.48 (1.19–5.15)	0.015
GI bleeding	4.15 (0.91–29.44)	0.092	2.25 (0.98–5.20)	0.057
Hepatopulmonary syndrome	NA (complete separation)	—	5.13 (1.48–17.71)	0.010
Pre-operative AST (per U/L)	1.00 (1.00–1.00)	0.391	1.00 (1.00–1.00)	0.037
(B)				
Variable	Logistic aOR (95% CI)	*p*-value	Cox aHR (95% CI)	*p*-value
Portal hypertension	7.41 (1.51–55.42)	0.023	3.63 (1.40–9.42)	0.008
Hepatopulmonary syndrome	NA †	—	10.23 (2.19–47.85)	0.003
GI bleeding	0.72 (0.04–19.87)	0.821	1.48 (0.48–4.56)	0.499
Variceal bleeding	2.93 (0.39–29.07)	0.303	0.74 (0.25–2.19)	0.584
Pre-operative AST (per U/L)	—	—	1.00 (1.00–1.00)	0.082

OR: odds ratio; HR: hazard ratio; CI: confidence interval. NA: hepatopulmonary syndrome excluded from logistic model (all three affected patients reached the transplant endpoint; complete separation). Only variables with *p* < 0.10 in either model shown. Denominators differ by variable owing to incomplete complication documentation (portal hypertension *n* = 65, variceal bleeding *n* = 64, gastrointestinal bleeding *n* = 68, hepatopulmonary syndrome n = 65, pre-operative AST *n* = 73). Variables with *p* < 0.10 in univariate analysis entered into multivariable models. aOR: adjusted odds ratio; aHR: adjusted hazard ratio. † Hepatopulmonary syndrome excluded from logistic regression (complete separation); retained in Cox model. Proportional hazards assumption verified using Schoenfeld residuals. HPS estimate based on *n* = 3 and must be interpreted with caution. Post-KPE complications were modelled as fixed (ever/never) covariates because onset dates were not consistently available; estimates may therefore be subject to immortal-time bias and should be interpreted as associations rather than time-dependent effects. Both multivariable models were fitted on the 60 patients with complete data for all covariates (30 transplant events); 14 patients were excluded owing to incomplete complication documentation.

## Data Availability

The datasets analysed during the current study are available from the corresponding author upon reasonable request.

## Supplementary Materials

The following supporting information can be downloaded at: https://www.mdpi.com/article/10.3390/jcm15166275/s1. Figure S1: Technical modifications in KPE, Figure S2: Step-by-step demonstration of the modified KPE technique.
