# Supplementary material for: Standardised Transplant-Orientated Kasai Portoenterostomy in a Combined Kasai and Transplant Programme: An 11-Year Single-Surgeon Series of 74 Consecutive Cases
Source: J Clin Med. 2026 Aug 13;15(16):6275. doi: 10.3390/jcm15166275 (PMC13513449; doi:10.3390/jcm15166275)
Supplement: Supplementary file 1 [file jcm-15-06275-s001.zip › jcm-4454827-supplementary.pdf]

Supplementary Materials

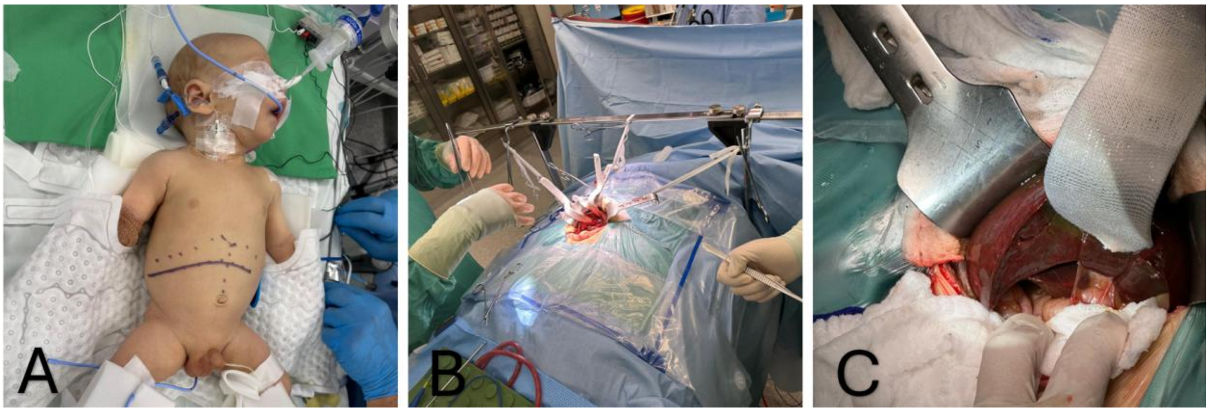

Supplementary Figure S1. Technical modifications in KPE. **(A)** pre-operative view showing the strategic placement of the transverse subcostal incision in a 4-month-old infant. The incision is positioned to align precisely with the standard transplant incision, eliminating the need for additional surgical access during subsequent transplantation **(B)** . Intraoperative photograph demonstrating the use of a Rochard retractor system that elevates the costal margin, providing optimal surgical exposure without requiring thoracoabdominal incisions. This approach minimizes adhesion formation in the chest cavity. **(C)** Intraoperative exposure demonstrating clear visualization of the hepatoduodenal ligament and porta hepatis achieved through the modified approach. Note the preservation of anatomical planes with the liver maintained in situ, avoiding mobilization that would complicate future hepatectomy. These modifications were applied uniformly across the series; the present study does not assess their effect on subsequent transplantation.

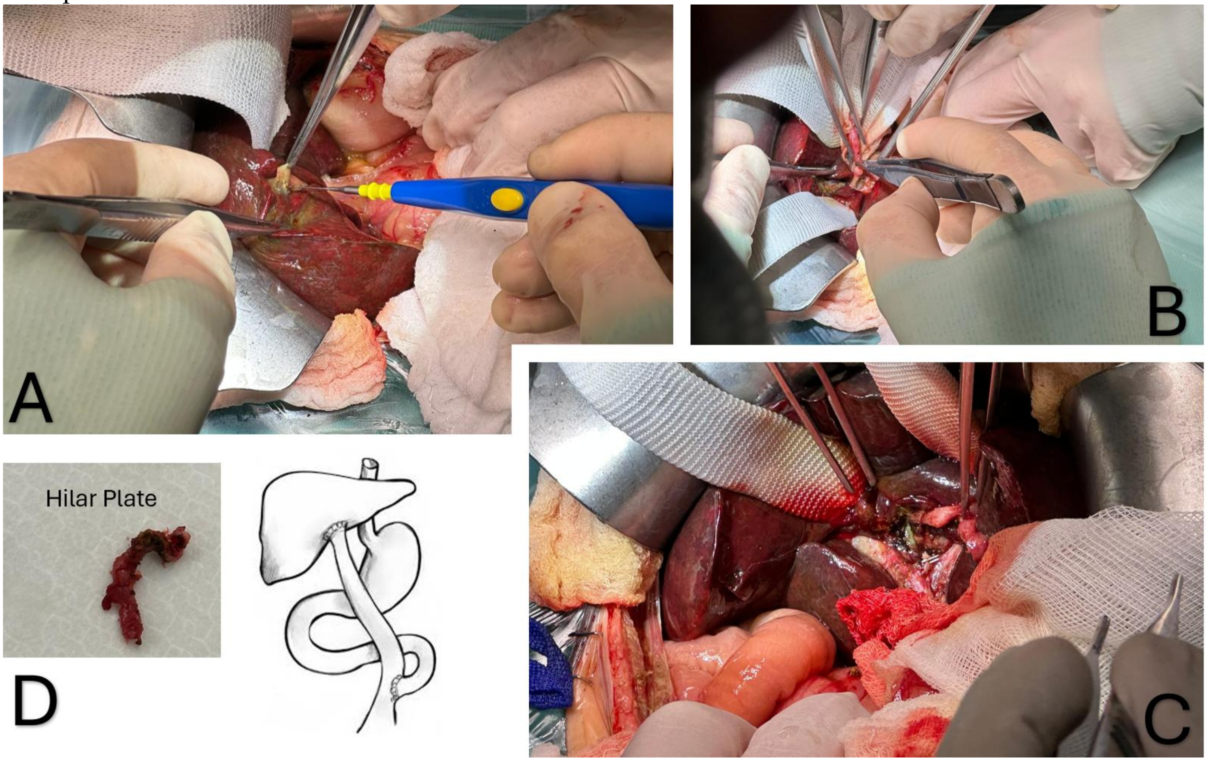

Supplementary Figure S2. Step-by-step demonstration of the modified KPE technique. **(A)** Initial dissection beginning at the obliterated atrophic gallbladder, which typically presents as a fibrotic cord due to early-onset biliary obliteration preventing bile flow. The electrocautery is used for precise

dissection while maintaining hemostasis. **(B)** Progressive dissection toward the hilar plate, exposing the fibrotic tissue that obliterates the bile ducts. Note the meticulous preservation of surrounding structures while working toward the porta hepatis. **(C)** Excision of the fibrotic biliary remnant and hilar plate tissue. The dissection extends into the liver parenchyma at the level of Glisson's capsule, carefully calibrated to uncover any patent bile canaliculi while avoiding excessive depth that could compromise hemostasis. The inset shows the excised hilar plate specimen, and the diagram illustrates the anatomical relationships of the Roux-en-Y reconstruction. **(D)** Final view after cystic plate dissection showing the prepared hepatic surface ready for portoenterostomy. The anastomosis is constructed between the exposed porta hepatis (with fibrotic rim posteriorly and Glisson's capsule anteriorly) and the Roux limb. The anastomosis is constructed between the exposed porta hepatis and the Roux limb
